# Partially brain effects of injection of human umbilical cord mesenchymal stem cells at injury sites in a mouse model of thoracic spinal cord contusion

**DOI:** 10.3389/fnmol.2023.1179175

**Published:** 2023-06-05

**Authors:** Haijun Hu, Houqing Long, Zhenxiao Ren, Tianhua Liu, Jinghui Xu, Fan Xiao

**Affiliations:** ^1^Department of Anesthesiology, The Second Affiliated Hospital of Nanchang University, Nanchang, Jiangxi, China; ^2^Department of Spine Surgery, Orthopaedic, Shenzhen People's Hospital (The Second Clinical Medical College, Jinan University/The First Affiliated Hospital, Southern University of Science and Technology), Shenzhen, Guangdong, China; ^3^Guangdong Provincial Key Laboratory of Orthopaedics and Traumatology/Department of Spine Surgery, The First Affiliated Hospital, Sun Yat-sen University, Guangzhou, Guangdong, China; ^4^Department of Oncology, Guangzhou Modern Hospital, Guangzhou, Guangdong, China

**Keywords:** spinal cord injury (SCI), contusion, human umbilical cord mesenchymal stem cells (hu-MSCs), anterior cingulate cortex (ACC), periaqueductal gray (PAG), neurogenic pain

## Abstract

**Purpose:**

The pain caused by spinal cord injury (SCI) poses a major burden on patients, and pain management is becoming a focus of treatment. Few reports have described changes in the brain after SCI. Particularly, the exact mechanism through which brain regions affect post-injury pain remains unclear. In this study, we aimed to determine the potential therapeutic mechanisms of pain. A mouse model of spinal cord contusion was established, and molecular expression in the anterior cingulate cortex (ACC) and periaqueductal gray (PAG) in the brain and animal behavior was observed after local injection of human umbilical cord mesenchymal stem cells (HU-MSCs) at the site of SCI.

**Method:**

Sixty-three female C57BL/6J mice were divided into four groups: a sham operation group (*n* = 15); a spinal injury group (SCI, *n* = 16); an SCI + HU-MSCs group (*n* = 16) and an SCI + PBS group (*n* = 16), in which the SCI site was injected with HU-MSCs/phosphate buffer. The BMS score was determined, and the von Frey test and Hargreaves test were used to assess behavior every week after surgery. Mice were sacrificed in the fourth week after operation, and samples were collected. The expression of CGRP, Substance P, C-Fos and KCC2 in the ACC and PAG were observed with immunohistochemistry. Chromic cyanine staining was used to observe transverse sections of the injured spinal cord.

**Result:**

In the ACC and PAG after SCI, the expression of CGRP, SP and C-Fos increased, and the expression of KCC2 decreased, whereas after HU-MSC injection, the expression of CGRP, SP and C-Fos decreased, and the expression of KCC2 increased. The SCI + HU-MSC group showed better exercise ability from 2 to 4 weeks after surgery than the SCI/SCI + PBS groups (*P* < 0.001). Local injection of HU-MSCs significantly improved the mechanical hyperalgesia caused by SCI in the fourth week after surgery (*P* < 0.0001), and sensation was significantly recovered 2 weeks after surgery (*P* < 0.0001); no improvement in thermal hypersensitivity was observed (*P* > 0.05). The HU-MSC group retained more white matter than the SCI/SCI + PBS groups (*P* < 0.0001).

**Conclusion:**

Local transplantation of HU-MSCs at the site of SCI partially relieves the neuropathic pain and promotes recovery of motor function. These findings suggest a feasible direction for the future treatment of SCI.

## 1. Introduction

Spinal cord injury (SCI) is a disease that destroys the central nervous system. SCI poses a substantial economic burden on patients and society, and therefore is a serious public health problem. Chronic moderate to severe pain, which is often disabling, occurs below the level of the SCI after injury, with an incidence as high as 53%−80% (Burke et al., 2017; Wu et al., 2020). In addition, hyperalgesia and heterologous pain are typical symptoms of SCI that severely affect patient quality of life (Mann et al., 2013). However, during the treatment of SCI, relatively little attention is paid to pain. Moreover, the relationship between changes in brain regions and pain, particularly after SCI, remains unclear. Traditional therapies, such as surgery and drugs, do not work well, thus hindering treatment (Finnerup et al., 2005). Therefore, relieving pain and improving patient quality of life remain important issues that must be solved in the treatment of SCI.

In recent years, stem cell transplantation, nanotechnology and other new technologies have been applied in the treatment of SCI and have achieved satisfactory results (Ren et al., 2023). Stem cell transplantation plays a major role in SCI repair. Stem cells are pluripotent cells that can multiply and differentiate into neurons, glial cells and other nerve cells under certain conditions, thus potentially facilitating SCI recovery (Wei et al., 2014; Ruzicka et al., 2017; Wu et al., 2020). The therapeutic effects of mesenchymal stem cells (MSCs) are associated with their secretory and supportive properties (Kaminska et al., 2022). MSCs promote functional recovery, angiogenesis and neurogenesis by producing and releasing exosomes in the brain, and can alleviate neuroinflammation (Baez-Jurado et al., 2019; Do et al., 2021). Human umbilical cord mesenchymal stem cells (HU-MSCs) have higher proliferation ability and lower risk of infection than stem cells from other sources. The immune response induced by local injection of HU-MSCs into injured tissues may also influence treatment (Zhang et al., 2013; Monguio-Tortajada et al., 2017). More evidence is needed to demonstrate that local injection of HU-MSCs is beneficial for the treatment of neuropathic pain in patients with SCI.

Recent studies on SCI have focused primarily on repair of the injury and the observation of the molecular changes in the injured area. In contrast, few studies have focused on the molecular changes in pain and brain areas. Pain pathways terminate in brain regions and are remarkably plastic (Kuzumaki et al., 2007).

The anterior cingulate cortex (ACC) is an area of the brain pain network believed to be involved in pain attention and emotional processing (Widerstrom-Noga et al., 2013). Emotional pain, physical pain and related cognitive control have been shown to be anatomically and functionally integrated in the ACC (Shackman et al., 2011). The ACC regulates pain and emotion by activating a variety of receptor systems [μ-opioid (Zubieta et al., 2001) and γ-aminobutyric acid (GABA) (LaGraize and Fuchs, 2007)] and the periaqueductal gray (PAG) (Widerstrom-Noga et al., 2013). Deep brain stimulation to the PAG has been found to decrease neuropathic pain in humans. Moreover, cannabis receptors, purinergic receptors, glutaminergic receptors, serotonergic receptors and adrenergic receptors in the PAG are believed to be associated with pain models (Voulalas et al., 2017).

The purpose of this study was to establish a mouse model of spinal cord contusion; examine the effects of local injection of HU-MSCs on molecular expression in the ACC and PAG in the brain; and conduct behavioral analysis of the mice to determine the effects of this treatment on neuropathic pain. Our findings may provide a research basis for stem cell therapy for neurogenic pain and diminished motor function caused by SCI.

## 2. Material and methods

### 2.1. Animals

**Figure 1:**
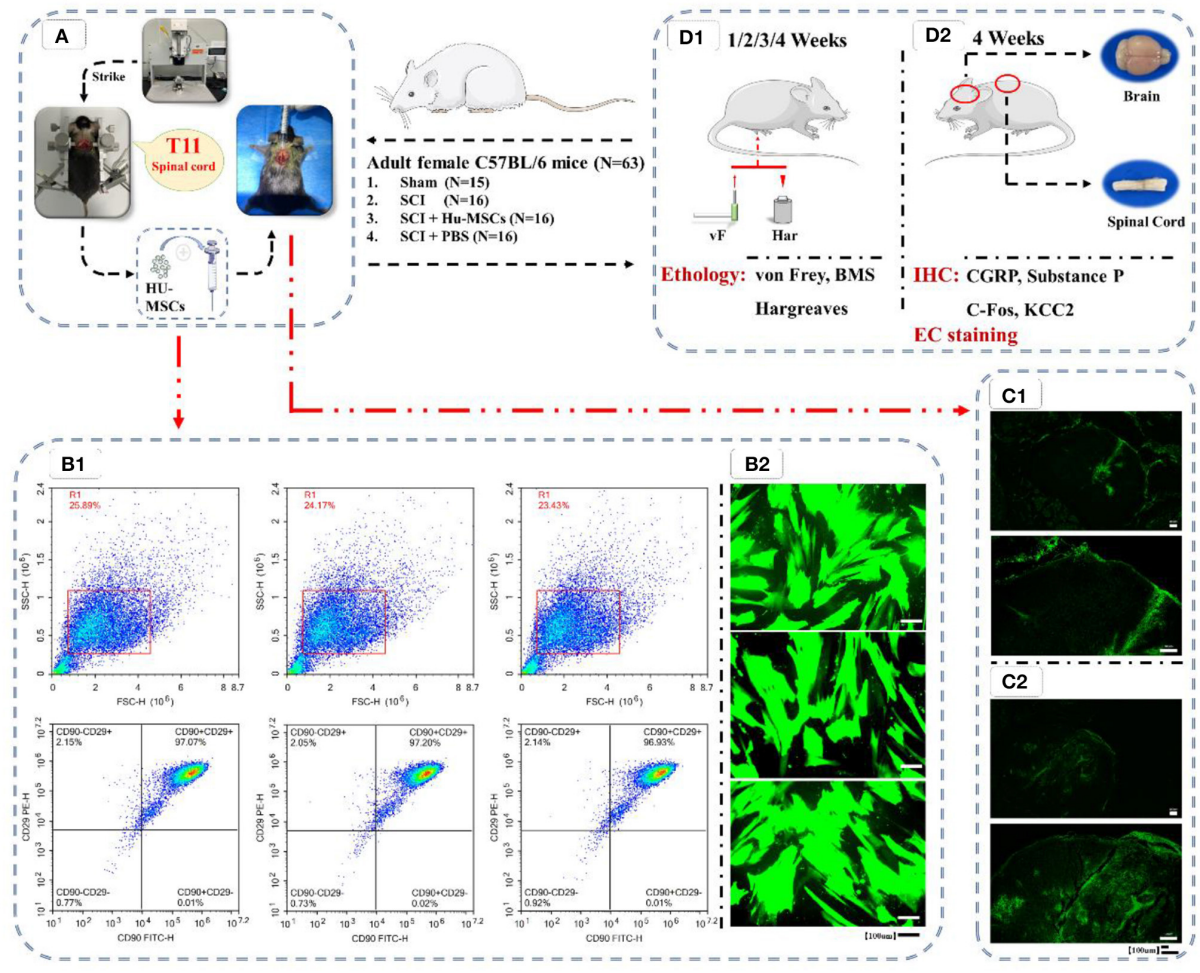
Flow chart of the experiment. **(A)** Preparation of animal models of T11 SCI and local injection of HU-MSCs. **(B1)** Flow cytometric analysis of HU-MSCs surface markers, and the cells were positive for CD29/CD90. **(B2)** The expression of GFP in the HU-MSCs was confirmed by microscopy, thus indicating that the plasmid was successfully introduced into the cells. **(C)** After injection of GFP-labeled stem cells in both the sham **(C1)** and SCI **(C2)** groups, the damaged area was observed by GFP staining 2–3 h later, thus indicating that HU-MSCs were correctly injected. **(D)** Behavioral **(D1)** and histological **(D2)** tests. **(D1)** Von Frey tests, BMS score calculation and Hargreaves behavioral tests were performed at 1, 2, 3, and 4 weeks after operation. **(D2)** At the fourth week after operation, mice were sacrificed, and brain and spinal cord samples were collected. The expression of CGRP, Substance P, C-Fos and KCC2 in the ACC and PAG in the brain were observed by immunohistochemistry (IHC). The cross-sections of injured spinal cords were observed through eriochrome cyanine (EC) staining.

A total of 63 C57BL/6J female mice [8 weeks of age; 20–25 g; SPF grade; Hunan Slyke Jingda Laboratory Animal Co., LTD., License No. SCXK (Hunan) 2019-0004]. The mice were housed in groups of three to five per cage under a 12/12-h light-dark cycle, and were given free access to food and water. The mice were divided into four groups: a sham operated group (*n* = 15); an SCI group (*n* = 16); an SCI + HU-MSC group (*n* = 16), with HU-MSCs injected at the site of SCI; and a SCI + PBS group (*n* = 16), with phosphate buffer solution injected at the site of SCI. All four groups of mice received surgery, behavioral testing and histological examination. The procedure was performed by two experimenters. After 1 week of acclimation, the mice were subjected to model induction. All procedures were approved by Jiangxi Zhonghong Boyuan Biotechnology Co. Ltd. (No. 2021011501; Figure 1).

### 2.2. Cell culture

#### 2.2.1. Cell culture and transfection

The supernatant of HU-MSCs (Zhong Hong Boyuan) culture was discarded, and cells were washed twice with 1 × Phosphate buffer saline (PBS). Then 0.25% pancreatic enzyme (containing 0.02% EDTA; T1300, Solarbio) was added, and digestion proceeded for 1–2 min. After the cells became rounded, Dulbecco's modified Eagle's medium [DMEM/F-12, GlutaMAX™ (10565018, Gibco)] was added to terminate digestion. The cell suspension was collected into a 10 mL centrifuge tube and centrifuged at 1,000 rpm for 3 min (TD4A, Changsha Yingtai). The supernatant was discarded, and DMEM was used to resuspend the cells. Cells were passaged 1:3 into new dishes, which were placed in a 37°C, 5% CO_2_ incubator (BPN-80CW, Shanghai Yiheng Scientific Instrument) for further culture. After culture, the cells were digested and counted as described above. The suspension was diluted and divided into Petri dishes for culture, and the cells were in good condition for transfection. Green fluorescent protein (GFP) lentivirus (GFP chronic viral empty vector; C8150, Zhong Hong Boyuan) transfection was then performed when the cells were in good condition.

After the cells were confirmed to be in good condition, the medium was changed to complete culture medium containing 5 μg/ml of the polybrene. A total of 2 × 10^5^ cells were seeded in each well of a six-well plate. According to the viral titer, a corresponding volume of lentivirus solution was added to each well for transfection with a MOI of 50 (MOI = viral titer × virus volume/number of cells). The medium was changed 16 h later, and the fluorescence intensity of cells was observed under an inverted fluorescence microscope (MF53, Guangzhou Mingmei Photoelectric) 72 h later (Figure 1B.2). Subsequently, normal HU-MSCs and HU-MSCs with GFP fluorescence were digested and counted, and 2 × 10^6^ cells were added to 10 μl PBS for resuspend, and injected into the mice.

#### 2.2.2. Flow cytometry

The identification of cell surface markers facilitates the identification and purification of specific cell types in research and clinical applications. Cell surface markers can be detected by flow cytometry. Cell surface markers like cluster of differentiation (CD) of MSCs were CD29, CD44, CD73, CD90, and CD105, but did not express CD14, CD34, CD45 and human antigen-DR (HLA-DR) (Ullah et al., 2015; Yip et al., 2020; Meyfour et al., 2021). In this study, the cells were identified as MSCs by flow cytometry detection of CD29/90.

Cells were digested and collected, then centrifuged to remove the supernatant. Subsequently, cells were washed with 1 ml PBS and centrifuged at 1,500 rpm for 5 min. The supernatant was discarded, and 50 μl PBS was added to resuspend the cells. Antibodies CD29 PE (303003, Biolegend) and CD90 FITC (328107, Biolegend) were added at 5 μl each. After light mixing, the cells were incubated at room temperature away from light for 20 min. Then 1 ml PBS was added, the cells were centrifuged at 1,500 rpm for 5 min, and the supernatant was discarded. Subsequently, 1 ml PBS was added, the cells were centrifuged at 1,500 rpm for 5 min, and the supernatant was discarded. Then 500 μl PBS was added and mixed to suspend the cells, and detection was performed with a NovoCyte™ flow cytometer [NovoCyte 2060R, Aisen Biology (Hangzhou); Figure 1B.1].

### 2.3. Surgical procedure

#### 2.3.1. Model preparation

After 1 week of acclimation, all mice underwent surgery to establish the SCI model. The mice were anesthetized by injection of a cocktail (2.5 ml/kg) containing ketamine (31.25 mg/kg; Dr. Ehrenstorfer), xylazine (1.58 mg/kg; TCL) and acepromazine (0.31 mg/kg; Meilunbio). After anesthesia, shaving, routine disinfection and surgical draping were performed. In the sham operation group, the skin and subcutaneous tissue were cut layer by layer, the paravertebral muscle was separated and exposed to the T9 vertebra, and the mice were sutured and sterilized. In the spinal cord contusion group, the skin and subcutaneous tissue were cut layer by layer, and the paravertebral muscle was separated to expose the target segment T9 vertebra. Subsequently the lamina was removed, and the spinal cord was exposed. The mice were fixed on an Infinite Horizon Impact Device (IH-0400 Impactor; Precision Systems and Instrumentation, Lexington, KY), and T8 and T10 were clamped with micro-forceps (Extra Fine Graefe Forceps; Fine Science Tools, Foster City, CA) to stabilize the spine. The impactor tip was adjusted 3–5 mm above the exposed spinal cord. The exposed spinal cord was then struck to induce moderate contusion (50 kdyn). Afterward, the mice were returned to the operating table. No extra operation was performed in the SCI group. The mice in the SCI + HU-MSC group were injected with HU-MSCs into the injured spinal cord, and the same amount of PBS solution was injected into the injured spinal cord in the SCI + PBS group. Then all tissues and skin were sutured. All mice were disinfected and returned to their cages. Lactate Ringer's solution (100 μl) was injected subcutaneously immediately after surgery to rehydrate. The retraction of the lower limbs and the spastic tail swinging were used as indicators of successful modeling.

#### 2.3.2. Model validation

To verify the proper injection of stem cells in the experimental group, we selected extra two mice not allocated to any group and performed sham/SCI model preparation according to the above steps and injection with GFP-labeled stem cells. The mice were sacrificed under deep anesthesia 2–3 h after injection, and the injured spinal cord was collected and stained for observation of GFP to determine whether the injection of stem cells had been performed correctly (Figure 1C).

#### 2.3.3. Post-operative care

The vital signs of the mice were observed closely after operation. the abdominal cavity was massaged twice per day to facilitate urination, and antibiotics were administered to prevent infection (ampicillin 33 mg/kg; Meilunbio) until reflexive bladder urination resumed. In the first 2 days after surgery, analgesics were administered (0.01 mg/kg, twice per day; Meilunbio). All surgeries and operations were performed by the same experienced physician.

### 2.4. Behavioral testing

Behavioral tests were performed on all mice weekly for 4 weeks postoperatively (Figure 1D). The mice were allowed 15–30 min to familiarize themselves with their environment before each test. All tests were performed by the same experienced researcher, who was blinded to the groups of the mice.

#### 2.4.1. Mechanical sensitivity

The Von Frey test was used to assess mechanical sensitivity in the mice. The tester consisted of a stimulus base, a metal shelf and plexiglass chambers. The plexiglass chambers (J&K Scientific) were located on the metal shelf, which was mounted on the stimulus base. During the test, we used von Frey filaments (Touch Test Sensory Evaluators; Gilroy, CA) with strengths of 0.16 and 1.4 g to stimulate the plantar surfaces of the hind paws. The left and right hind paw were stimulated five times each, and mechanical sensitivity was indicated by the percentage response rate to the mechanical stimulus, whereas hind limb contraction was identified as a positive response. The mechanical sensitivity of each mouse was determined by averaging the number of reflex contractions of both hind limbs. Three days before the surgery, we determined the baseline withdrawal frequency by separately measuring the withdrawal rate of filaments each day. The average of the first two preinjury values per animal and filament was used as the baseline withdrawal response rate. The mechanical sensitivity of the mice was assessed weekly for 4 weeks after surgery (Figure 1D.1).

#### 2.4.2. Thermal sensitivity

The Hargreaves method was used to test thermal perception in the mice. The plantar test (Ugo Basile Srl) emitted an infrared heat beam to stimulate the plantar region of both the left and right hind paws of the mice four times each. The latency period for withdrawal within seconds of the thermal stimulus was recorded. The intensity of the heat beam was then adjusted to baseline with a latency between 8–10 s (190 ± 1 mW/cm^2^). The cut-off time was set to 14 s to prevent paw injury. The order of detection of the hind paws on both sides in each mouse was randomized to avoid order-specific effects. Moreover, the mice were given a 1–2 min rest period between tests on the same hind paw. The data on both hind paws were then averaged to obtain the thermal sensitivity data for each mouse. Baseline measurements and processing times were the same as with the von Frey method.

#### 2.4.3. Locomotor function

To evaluate the recovery of motor function in the mice, we used the Basso Mouse Scale (BMS). A score of 0 indicated inability to perform ankle movements, whereas stepping movement occurred with a score of 3. A score of 4 indicated a plantar step. The front and hind limbs began to become coordinated at a score of 5, and highly coordinated at scores of 6 or 7. A score of 9 indicated normal movement. The scores of both hind limbs were calculated separately and averaged to obtain the BMS score for each mouse.

### 2.5. Tissue processing

Four weeks after surgery, the mice were sacrificed with an overdose of anesthesia cocktail. Saline (0.9%) was perfused into the heart to remove blood and was followed by 4% paraformaldehyde in 0.1 M phosphate buffer. The spine and brain were fixed in 4% paraformaldehyde for 1 h at room temperature, then rinsed with ddH_2_O and with 0.1 M phosphate buffer for 5 min. Samples were then cryoprotected in 30% sucrose in 0.1 M phosphate buffer at 4°C. The brain (ACC and PAG) and T11 spinal cord were identified according to anatomical landmarks, then embedded in Tissue-Tek O.C.T. compound (Sakura, America). Serial sections were then made (spinal cord, 25 μm coronal; brain, 25 μm) and mounted on slides (J&K Scientific). All samples from different groups were processed simultaneously, and sliced. Slides were stored at −80°C until staining and immunolabeling.

### 2.6. Eriochrome-cyanine staining

The myelin sheath was stained with eriochrome-cyanine (EC) to assess the size of the injured area by quantification of the non-injured tissue at the injury site. The slides were dried at 37°C for 2 h, then placed in fresh acetone for 5 min, removed and air-dried at room temperature for 20 min. Subsequently, staining with EC solution (0.4 g eriochrome cyanine R, 8 ml of 10% ferric ammonium sulfate solution, 1 ml concentrated H_2_SO_4_ and 192 ml ddH_2_O) was performed for 30 min at room temperature. After rinsing in ddH_2_O for 5 min, differentiation in 5% aqueous ferric ammonium solution in water was performed for 10–20 min until gray matter could be observed. The slides were rinsed in ddH_2_O and ferricyanide solution (2 g disodium tetraborate decahydrate, 2.5 g potassium ferricyanide and 200 ml ddH_2_O) for 10 min to complete differentiation. Subsequently, rinsing with ddH_2_O solution was performed for 5 min, and the tissue was dehydrated with a graded ethanol series (70%, 95% and 100%). The slides were then encapsulated. A microscope (BX43, OLYMPUS/CX43, OLYMPUS) was used to magnify each slide at the same resolution, lens aperture and exposure time. Quantification of cross-sectional area [CSA; as white matter area (WMA)/CSA] indicated loss of gray matter. Data analysis was performed in Image J Pro by a researcher who was blinded to the groups of mice.

### 2.7. Immunohistochemistry and quantitative analysis

Immunohistochemical labeling was performed on slides of thoracic spinal cord and brain serial sections. Dewaxing and hydration were performed after baking flakes [Electric blast Drying Oven (HGZF-101-1, Shanghai Yuejin Medical Equipment)] at 65°C for 2 h. Antigen retrieval solution and PBS were used for sliced antigen retrieval. Afterward, 3% hydrogen peroxidase was used to remove endogenous peroxidase, followed PBS flashed slides. Slides were then incubated with 0.5% Triton X-100 at room temperature for 20 min. The slides were soaked three times in PBS for 5 min each, then blocked for 30 min at 37°C with 5% BSA. Absorbent paper was used to blot the blocking solution without washing, and primary antibodies to the following were then added: Calcitonin gene-related peptide (CGRP; 1:100; DF8512, Affinity); K+-Cl-co-transporter (KCC2; 1:100; 19565-1-ap, Proteintech); Substance P (SP; 1:100; DF7522, Affinity); and C-Fos (1:100; AF5354, Affinity). Slides were placed in a wet box and incubated overnight at 4°C. The slides were subsequently removed and allowed to stand at room temperature for 45 min, then soaked with PBS three times for 5 min each. The slides were then incubated with horseradish peroxidase conjugated goat anti-rabbit IgG (H + L; 1:100; ZB-2301, ZSGB-BIO) at 37°C for 30 min, then washed with PBS. After DBA (CW0125, CWBIO) color development and hematoxylin (AR1180-1, BOSTER) counterstaining, the slides were sealed and subjected to microscopic examination. The microscope (BX43, OLYMPUS/CX43, OLYMPUS) was used to magnify each slide at the same resolution, lens aperture and exposure time. Images of each slide were obtained and analyzed. The specific staining intensity was evaluated in Image-Pro Plus 6.0 software (Media Cybernetics, Silver Spring, MD, USA). All images were obtained through an identical microscope and camera. The positive staining intensity of the cytoplasm and cell membranes was evaluated according to the mean integrated optical density (IOD) as follows: mean IOD = IOD/tissue slice area.

### 2.8. Statistical analysis

SPSS 20.0 software was used for statistical analysis. All experiments were repeated three times, and quantitative results are expressed as mean ± standard deviation (*X* ± SEM). The quantitative numerical comparison between groups was performed with independent sample *T*-tests, quantitative numerical comparison between multiple groups was performed with one-way ANOVA, and the S-N-K method was used for pairwise comparison (α = 0.05).

## 3. Results

### 3.1. Flow cytometry and cell transfection

The cells were analyzed by flow cytometry, and the cells showed positive results of (CD29/CD90), thus demonstrating that the cells were MSCs (Figure 1B.1). The expression of GFP in the HU-MSCs was confirmed by microscopy, which indicated that the plasmid was successfully introduced into the MSCs (Figure 1B.2). After injection of GFP-labeled stem cells in both the sham and SCI groups, GFP staining in the damaged area was observed 2–3 h later, thus indicating that the HU-MSCs had been correctly injected into the injury site (Figure 1C).

### 3.2. Behavioral testing

BMS score determination, and thermal (Hargreaves) and mechanical (von Frey) sensitivity tests were performed in the different groups of mice weekly after surgery. Motor and sensory recovery were evaluated with the behavioral testing described above.

#### 3.2.1. BMS score

**Figure 2:**
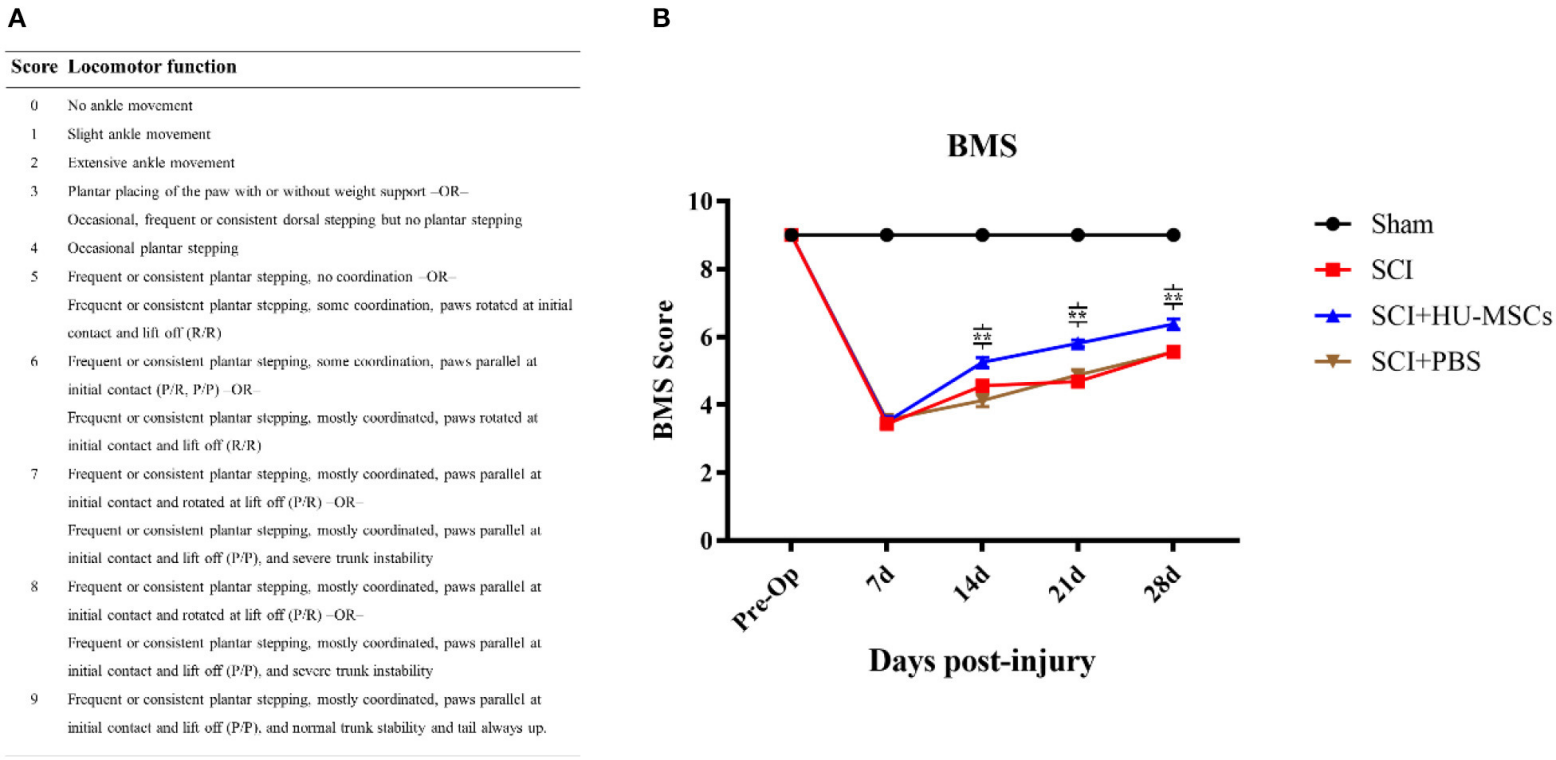
**(A)** Basso Mouse Scale for Locomotion (BMS). **(B)** BMS scores of mice in the different groups at 1, 2, 3, and 4 weeks after surgery. No difference was observed in the scores for the four groups 7 days after surgery, and the SCI + HU-MSC group showed relatively better exercise ability from 2 to 4 weeks after surgery (two-way ANOVA; 14, 21, 28 days, SCI + HU-MSC vs. SCI/SCI + PBS, **P < 0.001). The sham-operated group always had the highest score, and no difference was observed between the SCI and SCI + PBS groups. Notably, although the SCI + HU-MSC group achieved better recovery after the second week, the score increase trend was similar to that in the SCI and SCI + PBS groups.

Animals with SCI showed significant hindlimb motor function defects at all time points after surgery (two-way ANOVA; 7, 14, 21, 28 days, sham vs. SCI/SCI + PBS/SCI + HU-MSC, *P* < 0.001), and the sham-operated group maintained the same function as that before operation. The SCI animals showed varying degrees of minor motor recovery over time. No differences were observed in the scores of the four groups 7 days after surgery, and the SCI + HU-MSC group showed better exercise ability than the SCI/SCI + PBS groups from 2 to 4 weeks after surgery (two-way ANOVA; 14, 21, 28 days, *P* < 0.001). No significant difference was observed between the SCI and SCI + PBS groups (two-way ANOVA, *P* > 0.05), thus indicating that local injection of PBS was not effective at treatment. Notably, although the SCI + HU-MSC group achieved relatively better recovery after the second week, the score increase trend was similar to that in the SCI and SCI + PBS groups (Figure 2).

#### 3.2.2. Thermal sensitivity

**Figure 3:**
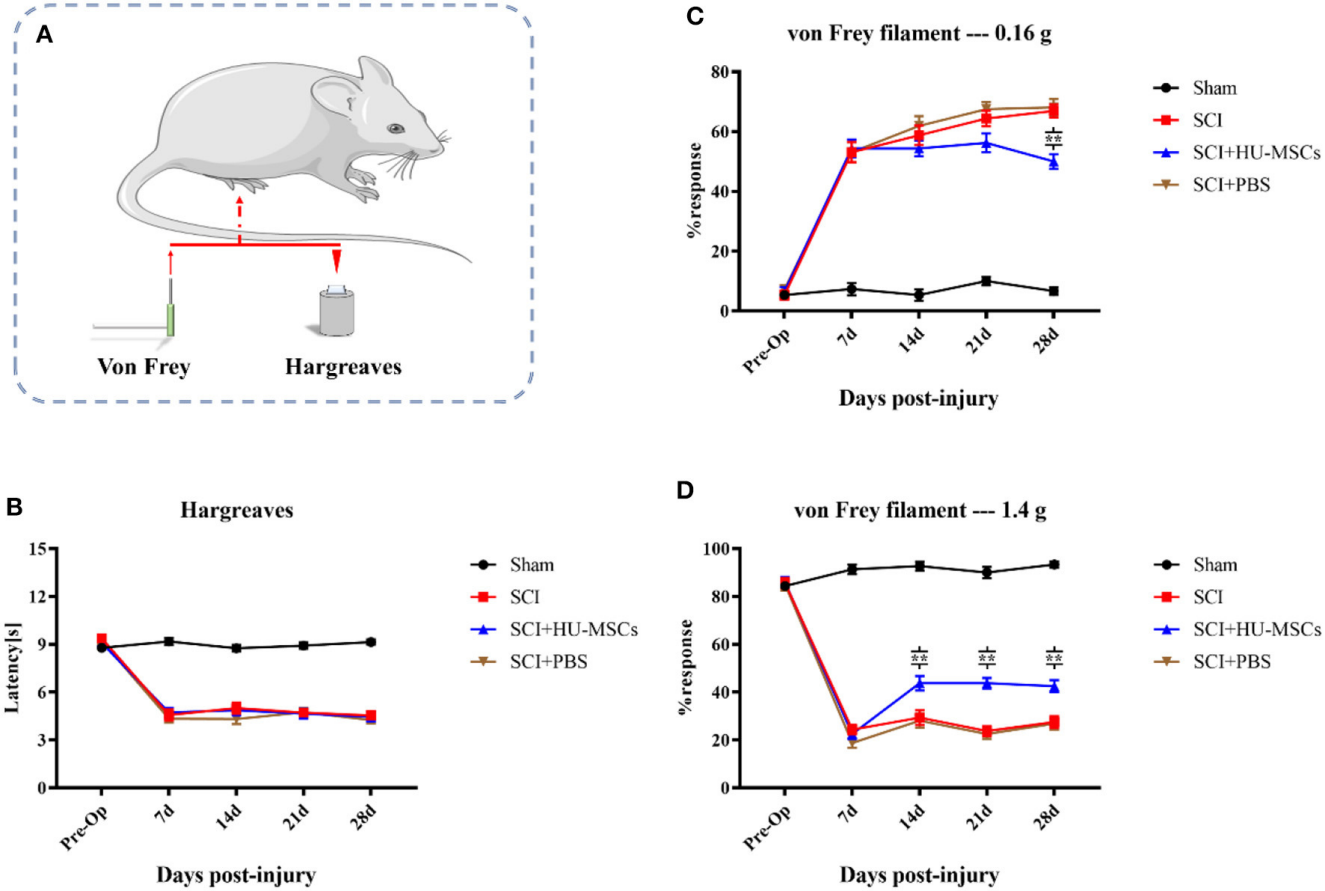
**(A)** Thermal (Hargreaves) and mechanical (von Frey) sensitivity tests were performed every week after surgery. **(B)** The sham-operated group always had the highest score, and no significant difference was observed among the other three groups (two-way ANOVA, *P* > 0.05). **(C)** When the plantar surfaces of the hind paws were stimulated with a 0.16 g von Frey filament, mice that had undergone SCI surgery developed allodynia. Local injection of HU-MSCs significantly ameliorated the mechanical hyperalgesia caused by SCI at the fourth week after surgery (two-way ANOVA; 28 d, SCI + HU-MSC vs. SCI/SCI + PBS, **P < 0.0001). However, no significant difference was observed at 7, 14, and 21 days after surgery. **(D)** The withdrawal response in the sham group was higher when a 1.4 g von Frey filament was used for heavy stimulation. No significant difference in withdrawal response was observed between the HU-MSC group and the SCI and SCI + PBS groups at 7 days post-operation. However, the withdrawal response in the HU-MSC group significantly increased after 14 days (two-way ANOVA; 14/21/28 days, SCI + HU-MSC vs. SCI/SCI + PBS, **P < 0.0001). These results suggested that local injection of HU-MSCs is useful for pain recovery after SCI.

All three groups of animals subjected to SCI showed significantly greater thermal hypersensitivity than the sham group (two-way ANOVA; sham vs. SCI/SCI + PBS/SCI + HU-MSC, *P* < 0.001). The sham-operated group maintained the same function as that pre-operation. At 7 days after injury, the withdrawal latency in all SCI animals decreased by 3–5 s from the baseline values. However, local injection of HU-MSCs and PBS appeared to have no restorative effect on thermal hypersensitivity in SCI animals (two-way ANOVA; SCI vs. SCI + PBS vs. SCI + HU-MSC, *P* > 0.05; Figures 3A, B).

#### 3.2.3. Mechanical sensitivity

When the plantar surfaces of the hind paws were stimulated with a 0.16 g von Frey filament, mice that had undergone SCI surgery developed allodynia. Local injection of HU-MSCs significantly ameliorated the mechanical hyperalgesia caused by SCI, as observed in the fourth week after surgery (two-way ANOVA; 28 days, SCI + HU-MSC vs. SCI/SCI + PBS, *P* < 0.0001). However, no significant difference was observed at 7, 14 and 21 days after surgery (two-way ANOVA; 7, 14, 21 days, SCI + HU-MSC vs. SCI/SCI + PBS, *P* > 0.05). Local injection of HU-MSCs decreased the withdrawal response of SCI animals to an intermediate level in the fourth week after surgery, thus indicating that MSCs partially resolved the allodynia caused by SCI. The withdrawal response in the sham group was higher when a 1.4 g von Frey filament was used for heavy stimulation. No significant difference in withdrawal response was observed between the SCI + HU-MSC group and the SCI and SCI + PBS groups at 7 days post-operation (two-way ANOVA; 7 days, *P* > 0.05). However, the withdrawal response of the SCI + HU-MSC group significantly increased after 14 days (two-way ANOVA; 14/21/28 d, SCI + HU-MSC vs. SCI/SCI + PBS, *P* < 0.0001). The heavy stimulation reflected the hyposensitivity of animals with SCI. The different responses among stimuli intensities suggested that the enhanced response of weak stimuli was caused by hyperreflexia and also can be used as an indicator of response to neuropathic pain. These results suggest that local injection of HU-MSCs aids in pain recovery after SCI (Figures 3A, C, D).

### 3.3. EC staining

**Figure 4:**
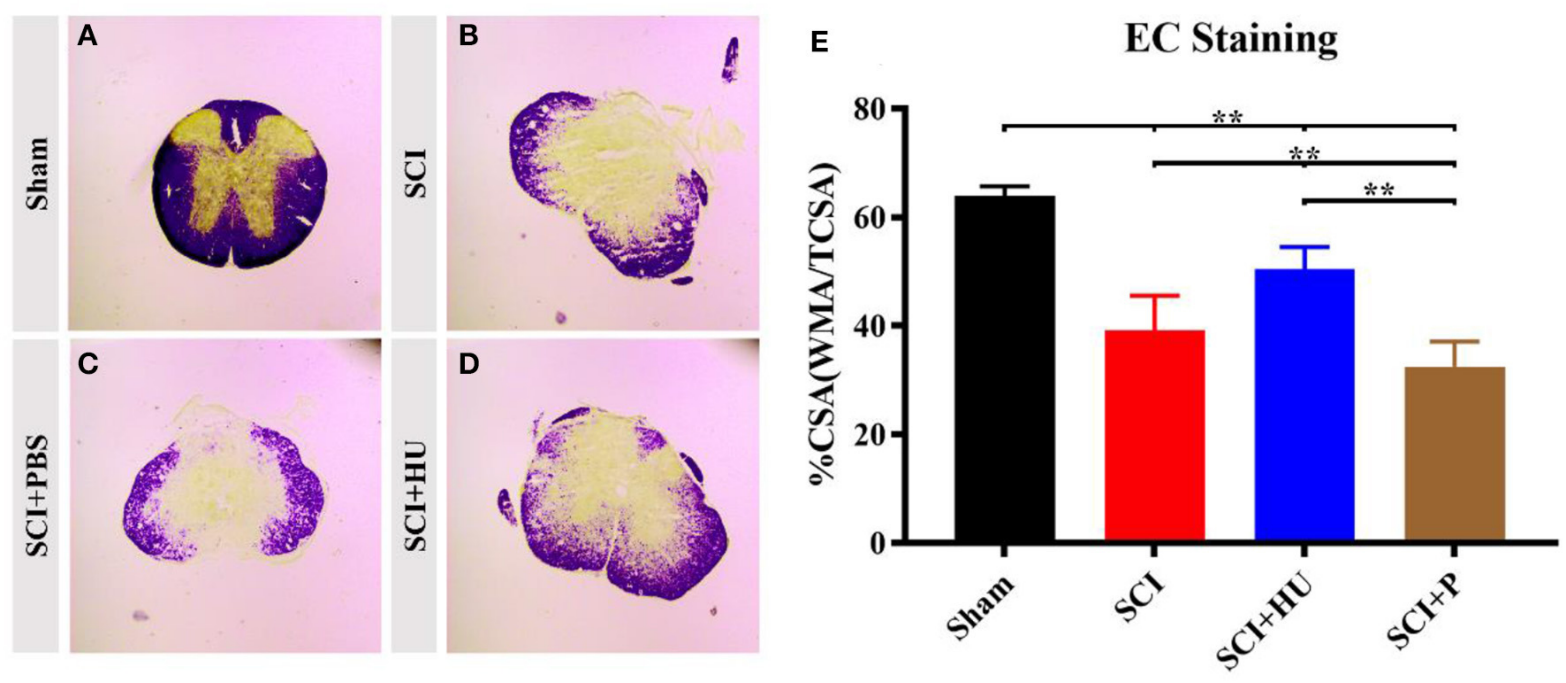
**(A–D)** Coronal sections of the spinal cords of mice in different groups were stained with eriochrome cyanine. Loss of gray-matter and a large amount of white matter were observed in the mice that underwent surgery. **(E)** Quantification of cross-sectional area (%CSA; WMA/CSA) indicates loss of gray matter. The results demonstrated that local injection of HU-MSCs affected the retention of white matter (one-way ANOVA; SCI + HU vs. SCI/SCI + PBS, **P < 0.0001).

To exclude the effects of differences in lesion size on pain sensitivity, we performed myelin staining (EC staining) on the coronal surface of the lesion. The differences in lesion size were then assessed through quantitative analysis of cross-sectional area (%CSA; WMA/CSA). Loss of gray-matter and a large amount of white matter were observed in the mice that underwent surgery. The %CSA in the SCI + HU-MSC group was significantly higher than that in the SCI and SCI + PBS groups (one-way ANOVA, ^**^P < 0.0001). The results demonstrated that local injection of HU-MSCs may have a certain effect on SCI repair (Figure 4).

### 3.4. Immunohistochemistry

**Figure 5:**
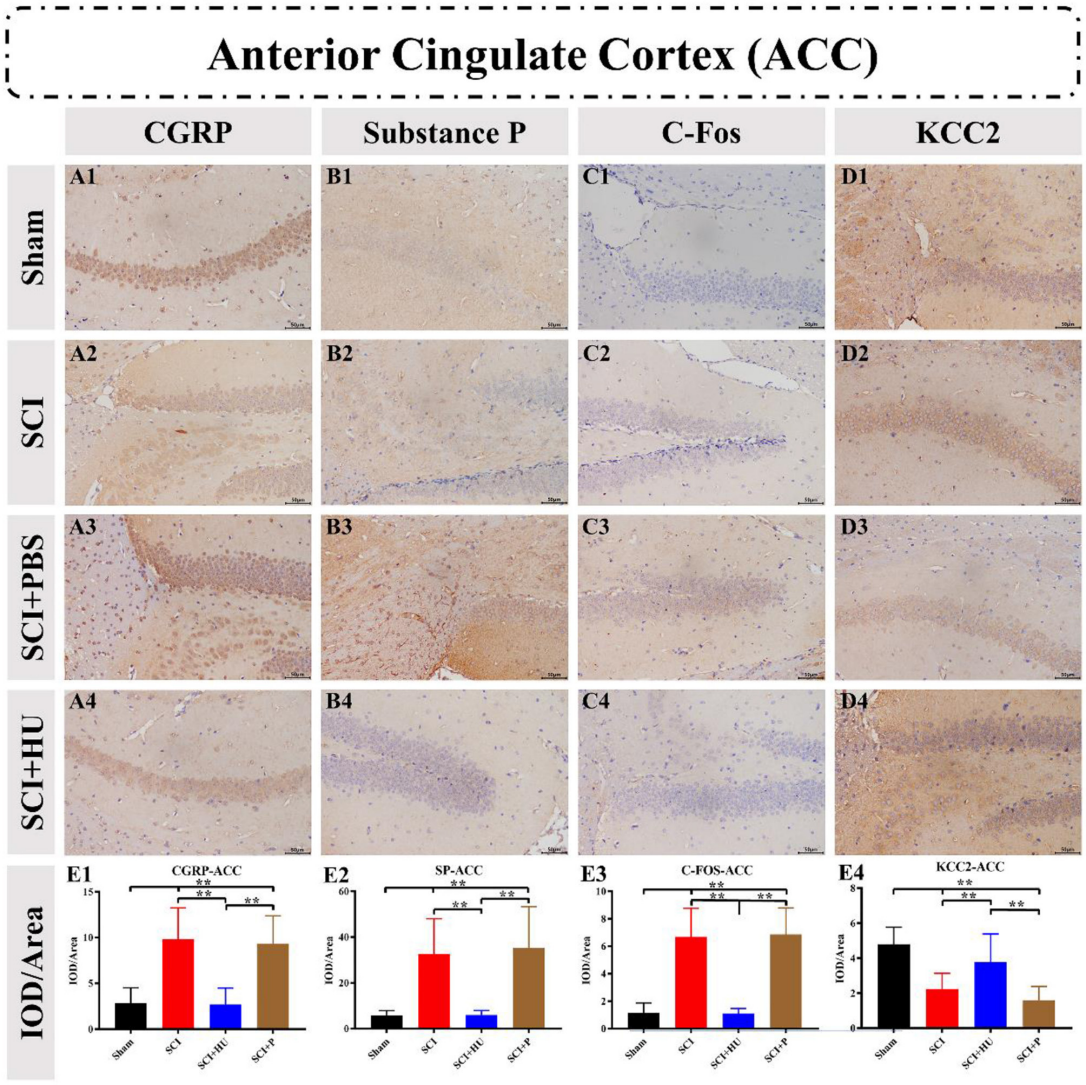
**(A–D)** Immunohistochemistry was used to detect the expression of CGRP, Substance P, C-Fos and KCC2 in the anterior cingulate cortex (ACC). **(E1–E4)** The integrated optical density (IOD) was quantitatively analyzed (IOD/area) to determine marker expression. The expression of CGRP, Substance P and C-Fos in the ACC in the SCI model increased, and the expression of KCC2 decreased (one-way ANOVA; SCI vs. sham, **P < 0.0001). The expression of CGRP, Substance P and C-Fos decreased, whereas that of KCC2 increased, after local injection of HU-MSCs (one-way ANOVA; SCI + HU-MSC vs. SCI/SCI + PBS, **P < 0.0001).

**Figure 6:**
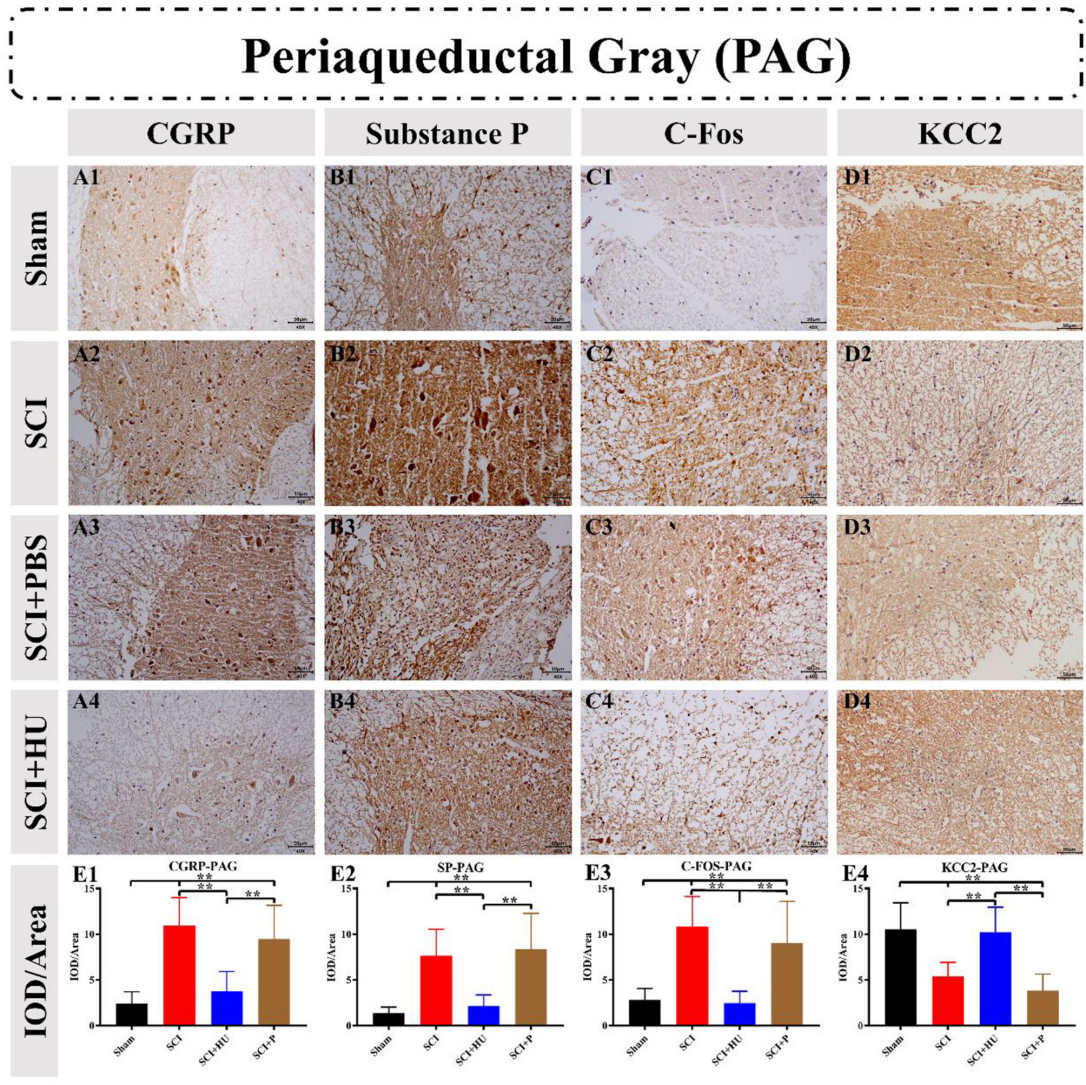
**(A–D)** Immunohistochemistry was used to detect the expression of CGRP, Substance P, C-Fos and KCC2 in the periaqueductal gray (PAG). **(E1–E4)** The integrated optical density (IOD) was quantitatively analyzed (IOD/area). The expression of CGRP, Substance P and C-Fos in the PAG in the SCI model increased (one-way ANOVA; SCI vs. sham, **P < 0.0001). The expression of CGRP, Substance P and C-Fos decreased, whereas that of KCC2 increased after local injection of HU-MSCs (one-way ANOVA; SCI + HU-MSC vs. SCI/SCI + PBS, **P < 0.0001).

Immunohistochemistry was used to detect the expression of CGRP, Substance P, C-Fos and KCC2 in the ACC and PAG. The IOD was quantitatively analyzed (IOD/area) to determine the expression of these markers. Compared with the sham-operated group, the expresion of CGRP, Substance P and C-Fos in the ACC in the SCI model increased, and the expression of KCC2 in the SCI model decreased (one-way ANOVA; SCI vs. sham, ^**^P < 0.0001). The expression of Calcitonin, Substance P and C-Fos decreased, whereas that of KCC2 increased, after local injection of HU-MSCs (one-way ANOVA; SCI + HU-MSC vs. SCI/SCI + PBS, ^**^P < 0.0001; Figure 5). In addition, the expression of CGRP, Substance P and C-Fos of the PAG in the SCI model increased (one-way ANOVA; SCI vs. sham, ^**^P < 0.0001). The expression of CGRP, Substance P and C-Fos decreased, whereas that of KCC2 increased after local injection of HU-MSCs (one-way ANOVA; SCI + HU-MSC vs. SCI/SCI + PBS, ^**^P < 0.0001; Figure 6).

## 4. Discussion

Diminished motor function and long-term moderate or severe pain below the injury plane caused by SCI remain major problems in clinical treatment. Because of the unclear mechanism and poor effects of traditional treatment methods, SCI treatment is very difficult. The brain is the center of pain processing, in which emotional pain, neuropathic pain and related emotional cognition are integrated in the ACC both anatomically and functionally. The ACC regulates pain and emotion partially through activating the PAG. However, the changes in these two regions after SCI remain unclear. In this study, lentiviral vector mediated GFP-labeled HU-MSCs were transplanted into the injured spinal cord in mice with spinal cord contusion, and the mechanism of pain after SCI was explored by observation of the changes in two brain regions, the ACC and PAG. This method had good effects in relieving neuropathic pain and restoring motor function, but not in treating thermal sensitivity. These findings might have been because, after local injection of HU-MSCs, stem cells diffused to the entire spinal cord and affected nerve function by participating in the regulation of the local microenvironment, and altering the expression of multiple proteins in the ACC and PAG.

After SCI, the structure of brain neurons and neuronal firing characteristics will change (Zhao et al., 2021). SCI changes the frequency and amplitude of sensorimotor neuron bursts. Cortical motor neurons regulate and compensate the synaptic strength and intrinsic properties of neurons, which may cause and maintain the overexcitation of cortical neurons, providing potential conditions for neuropathic pain. It is pointed out that the normal state of nerve point activity can be restored by external intervention of sensorimotor cortex, so as to achieve the purpose of regulating local brain function and relieving post-SCI pain (Keck et al., 2013; Zhao et al., 2021).

In recent years, new technologies, such as stem cell transplantation, nanotechnology and novel materials have been increasingly applied to the repair of SCI, thus providing new directions for the treatment of patients with SCI (Ren et al., 2023). Among them, cell transplantation is currently considered the preferred treatment for SCI. However, different stem cells have different fates, depending on the type and intrinsic properties of the transplant site (Su et al., 2012). Therefore, selecting stem cells from the proper source and transplanting them into the proper pathway at the proper time is the focus of SCI treatment. Both bone marrow mesenchymal stem cells (BMSCs) and HU-MSCs can relieve pain symptoms and promote motor function recovery in rats with SCI (Yousefifard et al., 2016). However, HU-MSCs are superior to BMSCS in terms of survival rates and electrophysiological data (Wei et al., 2012; Wu et al., 2020). After SCI, scar tissue can form over time, thereby hindering axon growth. Thus, stem cells should be transplanted early, 1–2 weeks after SCI, to decrease the effects of inflammatory factors and scar tissue on the growth of transplanted cells and axons (Wu et al., 2020). At present, the main cell transplantation methods include intravenous infusion transplantation and injury site transplantation. Intravenous infusion of stem cells is simple and carries little risk, but it does not ensure that the number of stem cells reaching the site of injury will attain therapeutic levels (Wu et al., 2020). Consequently, uncertain efficacy and potential high cost are the main disadvantages. However, local transplantation of stem cells at injury sites can accurately deliver stem cells into spinal cord tissue; this method is effective but may have a high complication rate. Studies have shown that intrathecal stem cell transplantation through lumbar puncture is effective and feasible in preventing and reversing pain and hyperalgesia caused by nerve ligation, as well as treating brain injury and other diseases (Chen et al., 2016; Hsuan et al., 2016). Moreover, clinical studies have shown that transplantation of mesenchymal stem cells can gradually relieve neuropathic pain in patients (Bao et al., 2018). Consequently, local transplantation of stem cells, or the combination of stem cells with nanotechnology and novel materials for the treatment of SCI, may effectively improve the outcomes of SCI surgery, increase safety and decrease the incidence of complications (Khan et al., 2018; Wu et al., 2020). Therefore, transplantation of HU-MSCs is beneficial for functional recovery after SCI, promotes repair of injured sites and improves motor function.

Thermal hyperalgesia, allogenic pain and hypoesthesia below the injury plane are typical symptoms of SCI (Sliwinski et al., 2018; Wu et al., 2020). Pain assessment in rodents relies primarily on stimulus-induced responses as a proxy for neuropathic pain, which are in fact an overreaction reflex. In a rat model of SCI, a local escape model has been used to assess pain levels after SCI (Sliwinski et al., 2018). When modified von Frey fibers (0.16 g) were used to stimulate mice with harmless intensity, a withdrawal response was induced, thus demonstrating hyperalgesia in SCI mice. Local transplantation of HU-MSCs decreased hyperalgesia behavior 4 weeks after injury. When von Frey fibers (1.4 g) were used for harmful stimulation, SCI mice showed hypoalgesia associated with SCI. At 14 days after injury, the group with local transplantation of HU-MSCs had more significant sensory recovery than the other groups. In thermal sensitivity tests with the Hargreaves method, the group with local transplantation of HU-MSCs did not show greater improvement in thermal sensitivity than the other injured groups. This finding may suggest that the mechanisms of hyperthermia and hyperalgesia after SCI are not the same as that in the repair pathway. In addition, the short observation time after injury might also explain why no repair of thermal hypersensitivity was observed. Further research is needed to explore the specific reasons for these findings. Neuropathic pain after SCI may be associated with local microenvironment changes, inflammation and neuronal protection (Wu et al., 2020), but the specific mechanism is still unclear. A decrease in inflammation may be the main reason for reducing pain associated with stem cell transplantation. Transplantation of stem cells has been shown to relieve pain by decreasing the activation level of inflammatory macrophages and microglia; inhibiting the expression of IL-6, IL-1β, IL-17A, TNF-α, iNOS, and 5-HT3A; and upregulating IL-10 (Zhilai et al., 2012; Bao et al., 2018; Wu et al., 2020). Stem cells have also been shown to up-regulate GDNF protein expression and promote vascular and functional recovery (Zhou et al., 2013; Wei et al., 2017). In addition, stem cells may enhance anti-inflammatory effects, anti-astrocyte proliferation, anti-apoptotic effects and axon preservation by promoting the polarization of M2 macrophages (Bao et al., 2018). The results of GFP and GFAP double staining of HU-MSCs exhibited good results, thus indicating that these cells have the potential for glial differentiation (Wu et al., 2020), and improve microcirculation and the microenvironment through therapeutic paracrine effects (Wei et al., 2014; Shiue et al., 2019), thus providing a basis for pain treatment in SCI.

In this study, we observed molecular expression in the ACC and PAG to determine the therapeutic effects of local transplantation of HU-MSCs on SCI. The ACC is an area of the brain's pain network believed to be involved in pain attention and emotional processing (Widerstrom-Noga et al., 2013). Moreover, emotional pain, physical pain and related cognitive control have been shown to be anatomically and functionally integrated in the ACC (Shackman et al., 2011). The ACC effectively regulates pain and emotion by activating the PAG. Deep brain stimulation of the PAG decreases neuropathic pain in humans (Voulalas et al., 2017). Our study indicated that the expression of CGRP, SP and C-Fos increased, and that of KCC2 decreased, in the ACC and PAG in models of SCI, whereas the expression of CGRP, SP and C-Fos decreased, and that of KCC2 increased, after HU-MSC injection. The molecules associated with pain were CGRP, SP and C-Fos. CGRP and its concomitant SP enhance the glutamate excitability of postsynaptic neurons, thereby affecting central sensitization caused by injury. Inhibition of signaling by the CGRP receptor, an important member of the subgroup of primary afferent pain receptors, alleviates the mechanical and thermal hypersensitivity at the incision, but has no effect on neuropathic pain. Therefore, CGRP in primary neurons appears to have different effects on pain caused by injury (Loken et al., 2021). SP is a neuropeptide that mediates pain signaling, and its accumulation is associated with ectopic nerve activity, thereby leading to neuropathic pain. SP binds its specific receptor, neurokinin-1 (NK-1), thus resulting in neuron sensitization and pain production. Decreasing SP expression through targeted therapy has been found to effectively relieve neuropathic pain (Manzhulo et al., 2016; Wang et al., 2020). The immediate early gene c-Fos is often used as a marker of neuronal activity in response to tactile, chemical, harmful and harmless stimuli. In many animal models of pain, tissue injury increases c-Fos expression, but the expression time is short, and the expression level returns to normal 1–3 weeks after injury. Fos can form the AP-1 transcription factor with a Jun dimer, thus upregulating tyrosine hydroxylase, enkephalin, proenkephalin, the 5-HT-2 receptor, the 5-HT-1A receptor and other genes involved in pain inhibition. C-Fos also upregulates the expression of CREBs associated with pain (Maeda et al., 2009). The expression of CGRP, SP and C-Fos in the HU-MSC treated group did not significantly differ from that in the sham operation group in the fourth week after injury, but was significantly lower than that in the SCI group. This finding indicates that neuropathic pain after SCI is associated with local microenvironment changes, and that, after local injection of HU-MSCs, stem cells diffuse to the entire spinal cord and participate in the regulation of the local microenvironment by changing the expression of local molecules, thus decreasing pain.

GABA inhibits neural activity in the adult central nervous system, inhibits excessive excitation and limits neural plasticity. However, the inhibitory effect of GABA on SCI can be decreased by down-regulating potassium/chlorine cotransporter (KCC2). Down-regulation of KCC2 increases chloride ions in the cell, thus decreasing hyperpolarization effects. The loss of GABA-dependent inhibition leads to spinal cord overexcitation, and consequently to abnormal motor activity and chronic pain. However, KCC2 activation restores the excitation-inhibition ratio of the spinal cord network after SCI; that is, the restoration of GABA inhibits and promotes the formation of relay neurons (Chen et al., 2018; Hudson and Grau, 2022). The use of the KCC2 activator CLP290 has also been shown to promote recovery of motor function (Chen et al., 2018). Our study indicated that the expression of KCC2 in the ACC and PAG regions of the brain decreased after SCI, but increased after treatment with HU-MSCs. The BMS scores in the treatment group were significantly higher than those in the SCI and SCI + PBS groups 14 days after injury. Our findings also demonstrated that local transplantation of HU-MSCs significantly affects motor function recovery after SCI. Changes in brain regions that control pain after SCI and the efficacy of stem cells in treating pain were investigated. However, the specific molecular mechanism through which stem cells affect pain and motor changes after SCI has not been explored, and, future studies are necessary to further examine its mechanism.

## 5. Conclusion

In the current study, we found that local transplantation of HU-MSCs partially relieved neuropathic pain caused by SCI and promoted the recovery of motor function. This effect may be associated with stem cells diffusing throughout the spinal cord, participating in local microenvironment regulation, and influencing neural function by altering the expression of multiple proteins in the ACC and PAG brain regions. Although the molecular mechanism underlying this phenomenon has not yet been fully explained, our findings provide a feasible direction for SCI pain treatment in the future.

## Data availability statement

The original contributions presented in the study are included in the article/supplementary material, further inquiries can be directed to the corresponding author. The raw data can be found at: https://www.jianguoyun.com/p/DbgHxIEQm9a5Cxj7_PgEIAA.

## Ethics statement

The animal study was reviewed and approved by Experimental Animal Welfare and Ethics Committee of Jiangxi Zhonghong Boyuan Biotechnology Co., Ltd.

## Author contributions

HH: animal experiment and manuscript writing. ZR: manuscript writing and revision and figures formulating and revision. HL: manuscript writing and revision and figures revision. JX and TL: manuscript revision. FX: experimental design, figures, and manuscript revision. All authors contributed to the article and approved the submitted version.
